# High pressure die casting of Fe-based metallic glass

**DOI:** 10.1038/srep35258

**Published:** 2016-10-11

**Authors:** Parthiban Ramasamy, Attila Szabo, Stefan Borzel, Jürgen Eckert, Mihai Stoica, András Bárdos

**Affiliations:** 1 IFW Dresden, Institute for Complex Materials, Helmholtzstr. 20, D-01069 Dresden, Germany; 2 Breuckmann GmbH & Co KG Heiligenhaus, Germany; 3 Budapest University of Technology and Economics, Dept. of Auto. and Vehicle Manufacturing, Stoczek str. 6, H-1111 Budapest, Hungary; 4 TU Dresden, Institute of Materials Science, D-01062 Dresden, Germany; 5 Politehnica University Timisoara, P-ta Victoriei 2, RO-300006 Timisoara, Romania

## Abstract

Soft ferromagnetic Fe-based bulk metallic glass key-shaped specimens with a maximum and minimum width of 25.4 and 5 mm, respectively, were successfully produced using a high pressure die casting (HPDC) method, The influence of die material, alloy temperature and flow rate on the microstructure, thermal stability and soft ferromagnetic properties has been studied. The results suggest that a steel die in which the molten metal flows at low rate and high temperature can be used to produce completely glassy samples. This can be attributed to the laminar filling of the mold and to a lower heat transfer coefficient, which avoids the skin effect in the steel mold. In addition, magnetic measurements reveal that the amorphous structure of the material is maintained throughout the key-shaped samples. Although it is difficult to control the flow and cooling rate of the molten metal in the corners of the key due to different cross sections, this can be overcome by proper tool geometry. The present results confirm that HPDC is a suitable method for the casting of Fe-based bulk glassy alloys even with complex geometries for a broad range of applications.

After the discovery of the first metallic glass by Duwez and co-workers in 1960 by rapid quenching of an Au_80_Si_20_ liquid, in the beginning of 1980s^1^,^2^,^3^ bulk metallic glasses (BMGs) were produced successfully. Since then, a large number of BMGs was explored in a variety of multicomponent alloy systems^4^,^5^,^6^,^7^,^8^,^9^,^10^,^11^,^12^,^13^. The first Fe-based BMG has been synthesized in 1995^14^,^15^ by Inoue *et al*. Since then, many Fe-based BMGs have been developed and attracted a great deal of attention due to their superior properties such as high strength and good magnetic properties^16^,^17^,^18^,^19^,^20^,^21^,^22^,^23^, abundant natural resources and low material cost. Owing to the very high strength, brittleness^24^ and moderate glass-forming ability (GFA) of Fe-based BMGs, fundamental and application-oriented research on Fe-based BMGs was confined to wires, ribbons, rods and powder metallurgy (P/M)-based products^25^. Nowadays more and more applications require the promising mechanical and magnetic properties of bulk glassy alloys^25^, but large-scale manufacturing of the material is still a challenging issue and not solved, yet. With a few rare exceptions, mostly ribbons are produced at industrial scale^25^. Recently, Zhang *et al*.^21^ and Wu *et al*.^18^ reported on the direct fabrication of toroidal cores from Fe_66_Co_10_Mo_3.5_P_10_C_4_B_4_Si_2.5_ BMG, having an inner diameter of 6 mm and an outer diameter of 10 mm. There have been only a few reports on the direct fabrication of bulk magnetic cores using casting^26^. The reason is simple: the Fe-based BMGs are very brittle and cannot withstand the industrial casting process, even if the material is a good glass former. Hence, so far casting and handling of bulk Fe-BMG samples are only limited to the laboratory scale. The most commonly applied process other than casting is powder metallurgy, but this route involves hot pressing or (spark plasma) sintering which typically deteriorate the soft magnetic properties of the glass^27^,^28^. Alternatively, thermoplastic forming (TPF) has also been used to fabricate three dimensional BMG parts^29^. In this method the glass is heated to its supercooled liquid regime (SLR) [.i.e. to temperatures between the glass transition temperature (*T*_*g*_) and the crystallization temperature (*T*_*x*_)]. In the supercooled liquid regime the viscosity of the glass decreases drastically^30^,^31^, which allows thermoplastic forming of the material. Even though this process offers near-net-shape geometry parts, very good surface finish and the ability to reproduce even very fine and small structures^32^,^33^,^34^,^35^,^36^, the processing time (*t*_*p*_) must be very short, in order to avoid crystallization. The short processing time is a major drawback in using this technique for producing bigger and complex parts. Other emerging additive manufacturing techniques, like selective laser melting (SLM), have also been used to produce BMG parts with refined microstructure and excellent properties^37^,^38^,^39^. However, this method requires uniformly-sized glass-forming alloy powders, which is very expensive and highly complicated to produce.

The current industry demands materials with low cost and improved mechanical properties: higher hardness, higher tensile strength and better surface qualities. As a general rule, a higher strength and hardness of the material brings along higher manufacturing costs and also higher manufacturing times, which limits their use. The purpose of our work is to design and to develop a high-pressure die casting method and suitable tools to directly cast Fe-based bulk metallic glasses, which satisfy the above mentioned requirements.

## High Pressure Die Casting (HPDC)

High pressure die casting (HPDC) has been widely used to manufacture a large variety of products with high dimensional accuracy and productivity^40^. It allows much faster production compared with other casting techniques and is an economical and efficient method for producing components requiring low surface roughness and high dimensional accuracy^41^. Table 1 summarizes the characteristics of all three processes, i.e. HPDC, TPF and SLM. It is clear that is not possible to conclude which process is the best, in most of the cases the final result depends on several aspects. However, for the actual case of Fe-based BMG forming alloy, the HDPC process has a number of advantages.

In our present work we designed a high pressure die casting tool (key-shaped) for casting Fe-based BMG and optimized the casting parameters for a Fe_74_Mo_4_P_10_C_7.5_B_2.5_Si_2_ alloy. This particular Fe-based composition was chosen because of its relatively low melting point of 1283 K, good glass-forming ability (GFA) and good soft magnetic properties^42^. This alloy can be produced from industrial grade elements, which makes it cheap and a realistic candidate for applications. Another advantage is its relatively low affinity towards oxygen, which makes it much easier to handle compared to Ti-based or Zr-based alloys.

## Experimental Details

### Sample Preparation and Characterization

The master alloys with nominal composition of Fe_74_Mo_4_P_10_C_7.5_B_2.5_Si_2_ were prepared by induction melting of Fe and Mo lumps (99.9 mass%), crystalline B (99.5 mass%) and crystalline Si (99.99 mass%). For C and P pure graphite and an industrial FeP pre-alloy with a P content of 26.9 at.% were used (the oxygen content of industrial FeP pre-alloy is around 480 ppm). Induction melting was preferred in order to assure a good homogeneity of the entire master alloys. Pieces of the master alloys were remelted in aluminum oxide crucibles. Subsequently, the molten metal was poured into the shot sleeve of the high pressure die casting machine and shot into the mold under a high purity Ar atmosphere (99.9% pure), in order to produce key-shaped specimens. The samples were cast in an industrial OGSZF110T vertical high pressure die casting machine. In laboratory conditions copper mold is used to cast up to 4 mm diameter rods by injection casting method. The thermal stability behavior of the glassy samples was evaluated with a NETZSCH DSC 404C differential scanning calorimeter (DSC) under flowing high purity argon at a constant heating rate of 20 K/min. The structural characterization of the as-cast amorphous key samples, as well as of crystallized samples, was performed by X-ray diffraction (XRD) using a PHILIPS PW 1050 diffractometer with Co-Kα radiation (λ = 0.17889 nm). Microstructure characterization was done using a Leica stereoscan 410 scanning electron microscope (SEM). For magnetic measurements, DC *M-H* hysteresis loops were measured with a vibrating sample magnetometer (VSM) at ambient temperature. The coercivity was measured using a Foerster Coercimat-type device under a DC magnetic field, which can be continuously changed from −250 mT to +250 mT. The accuracy of the measured data lies within ±2.5 K in the case of DSC measurements, ±0.1 A/m for coercivity and ±80 A/m (~1 Oe) for the VSM measurements.

### Tool Design - Modelling and Simulation

**Figure 1:**
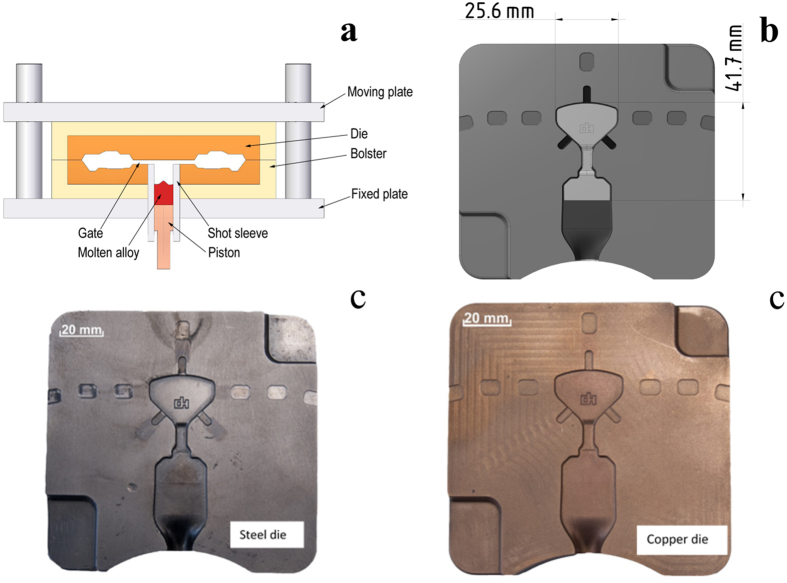
(**a**) Schematic illustration of the high pressure die casting setup; (**b**). 3D model of the die; (**c**). Completed dies made from heat resistant steel and a copper alloy.

A schematic drawing of the high pressure die casting (HPDC) method used in our experiments is shown in Fig. 1(a). In typical HPDC machine is normally composed of two major sections: a fixed section (lower half of the die) and a moveable section (upper half of the die and piston). Die temperature during the whole casting process must be monitored and accurately controlled, as the quality of the casting is very sensitive to the variation in temperature. When the molten alloy is in the die cavity, the heat in the alloy should be removed to allow solidification and subsequent cooling to occur. However, the die temperature depends on a number of process variables such as cycle time, spray distribution and duration, water layout and flow rate, casting volume/geometry, as well as molten metal temperature and composition. A fluid simulation was performed using the Flow3D simulation software to determine the minimum required filling speed and to optimize the casting tool geometry. Due to the insufficient knowledge of the main physical properties of the alloy which are necessary for casting, an earlier laboratory mold casting test was simulated. We were able to determine the melt front solidification time and also the form filling time at known casting parameters according to the simulation results. The results of the previously simulated mold casting tool were taken into account during the high pressure die casting simulation. During the high pressure die casting tool simulation a constant viscosity was chosen at the liquidus temperature as a boundary condition, to be sure that the alloy is able to fill up the mold cavity in a given time, before the solidification starts.

In the simulation an adiabatic filling process was assumed because of the high filling speed; the filling speed is about 15–20 ms (calculated solidification time ≈3.5*(typical wall thickness) ^2.06^ ms). According to the simulation results and the estimated cooling speed (calculated from empirical equations) the casting tool was designed and manufactured using high performance hot forming materials. The VISI modelling software was used to model the complete tool.

Figure 1(b,c) show the 3D model of the die and the completed die made from a copper alloy and heat resistant steel, respectively. Two different die materials were used for the casting purpose: Heat resistant steel with a low heat conduction rate of 33 W/mK and a Cu alloy with high heat conduction rate of 230 W/mK respectively. The reason for the choice of the two different die materials was to test the influence of heat conduction on glass formation and on the die life.

The die life in HPDC process depends on several factors but the very important factors are casting temperatures and hardness of the casting material. In casting of Al alloys single copper die can be used for around 100,000 castings- casting temperature and product hardness are low. However, in case of brass alloys only 5 to 6,000 samples can be cast using a single die (casting temperature and product hardness are relatively much higher).

In the case of this Fe-base BMG we have cast only 50–75 samples and we did not see any damage to the die. In the positive side the maximum casting temperature of this Fe-Mo-P-C-B-Si glass is around 100–120 K more than the brass alloy casting temperature but the hardness of the glass is 945 ± 5 HV, which is almost 4 to 5 times higher than the brass alloys. Hence we expect the die life to be around 3 to 4,000 castings.

## Results

**Figure 2:**
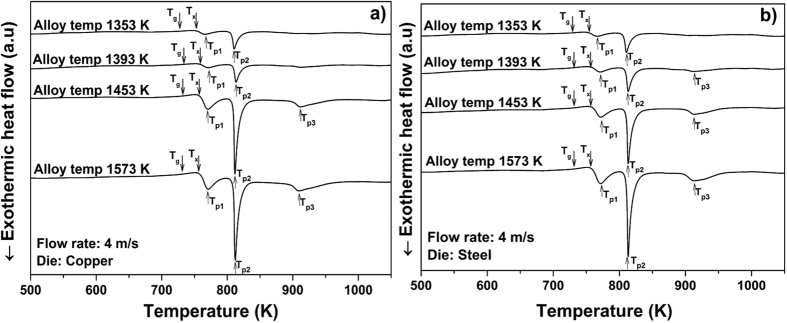
DSC curves (heating rate 20 K/min) of the glassy specimens cast at different temperatures using (**a**) copper die and (**b**) steel die.

**Figure 3:**
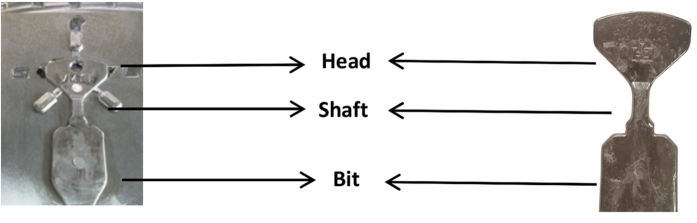
(**a**) Cast part (key) still in the mold cavity (**b**) Cast part (key) separated from the mold.

Figure 2(a,b) compare the DSC curves of the key-shaped parts cast at different temperatures (1353, 1393, 1453 and 1573 K, respectively) using both the copper and the steel die, with a constant flow rate of 4 m/s. In all cases the head of the key (see Fig. 3(a,b)) was used for characterization, because the molten metal travels the maximum distance and also flows through different cross sections, before reaching the key head. As a result the head part has a maximum chance for crystallization; hence this part is most critical and was chosen for the analysis.

**Figure 4:**
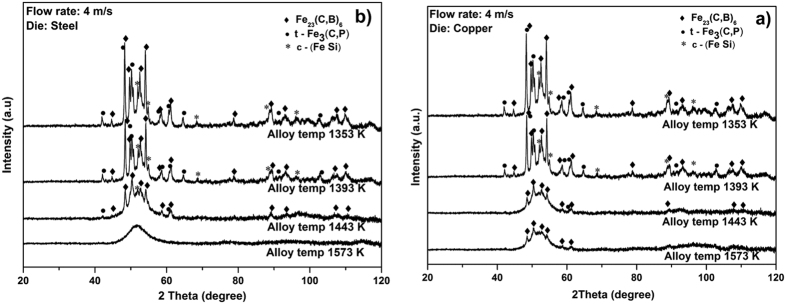
XRD patterns of the glasses cast at different temperatures using (**a**) copper die and (**b**) steel die.

A constant flow rate of 4 m/s (in case of higher flow rates the Cu die is damaged) was used for both dies for the whole range of casting temperatures investigated in order to have uniform casting conditions. The keys cast at higher temperatures (1573 K) clearly show three distinct crystallization peaks (*T*_*p*1_, *T*_*p*2_ and *T*_*p*3_), whereas for the keys cast at lower temperatures (1353 and 1393 K) the first (*T*_*p*1_) and third (*T*_*p*3_) crystallization peaks are not clearly visible. Table 2 compares the crystallization enthalpies for the first and second crystallization steps for the key samples cast at different temperatures using copper and steel dies with the sample cast in lab condition. The key samples cast at 1573 K exhibit almost the same first and second crystallization enthalpy values (−15.6 J/g and −38.8 J/g, respectively) as that of the sample prepared in lab condition. The other keys cast at the same temperature using the copper die and also the specimens cast at lower temperatures using both copper and steel dies show relatively low enthalpy values (see Table 2), indicating the presence of a small amount of crystals in the cast parts. These enthalpy values are in good agreement with the XRD data (see Fig. 4a,b). Except for the keys cast at high temperature (i.e. 1573 K) using the steel die, all other keys show crystalline peaks and the number and intensity of the crystalline peaks decrease with increasing casting temperature. However, the keys cast at 1573 K using the Cu mold still show some small crystalline peaks, indicating that the cooling rate was not sufficient to suppress the nucleation of crystals. This may be due to the formation of cold spots because of localized fluctuations followed by heterogeneous nucleation, which will be discussed later. Table 3 compares the values of the characteristic temperatures obtained for the key samples, the 3 mm rod samples cast in lab condition with the values from the literature.

**Figure 5:**
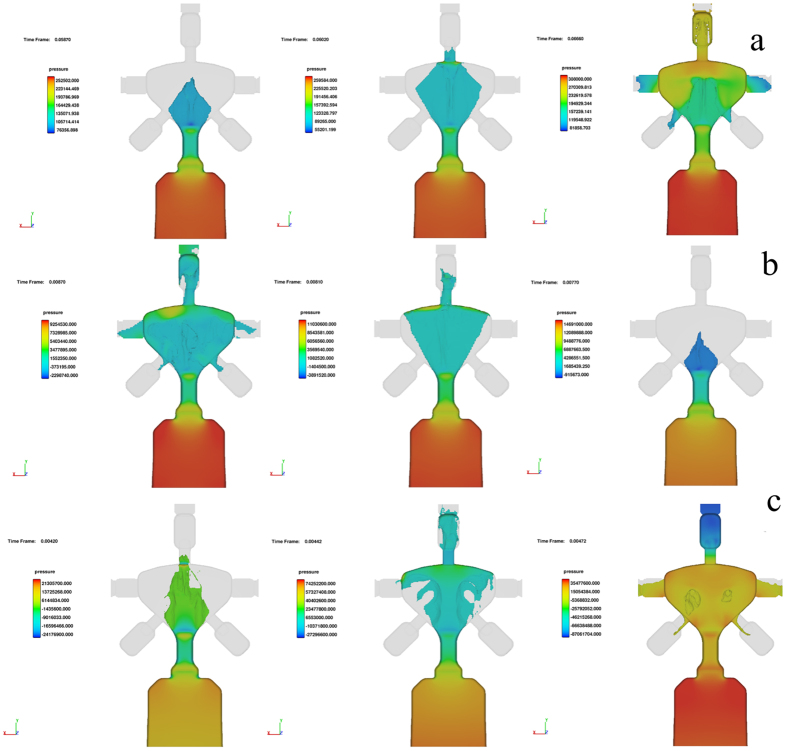
Flow3D simulation for three different flow rates: (**a**) 4 m/s, (**b**) 12 m/s and (**c**) 19 m/s.

The effect of the flow rate of the molten metal upon casting into the steel mold was studied by varying the piston speed. Figures 5a,b,c show the Flow3D simulation results for three different flow rates of 4, 12 and 19 m/s, respectively. For all simulations reported here, the liquid alloy temperature was maintained at 1523 K, due to the limited availability of reliable viscosity data for this alloy^43^. The legend gives the information about the pressure variation in the liquid metal flow at different cross-sections; red and blue color indicate the maximum to lowest pressure, respectively. The maximum laminar filling^40^ of the mold is obtained for a flow rate of 4 m/s (Fig. 5a), which is in good agreement with the DSC and XRD results for both copper and steel dies (see Figs 2a,b and 4a,b). When the flow rate increases to 12 m/s the flow becomes turbulent during the last stage of filling, and when the flow rate further increases up to 19 m/s the flow becomes even more turbulent. The key-shaped samples cast at 12 and 19 m/s exhibit a crystallization enthalpy of −13.1 and −9.8 J/g, respectively, during the first step of crystallization. These values are significantly lower compared with the crystallization enthalpy found for the key samples cast at a flow rate of 4 m/s (i.e. −15.6 J/g). This reduced glass formation of the alloy with increasing flow rate upon HPDC may be due to strongly pronounced viscosity shearing occurring during fast mold filling, i.e. at high flow rates, which increases the fragility of the glass^44^.

**Figure 6:**
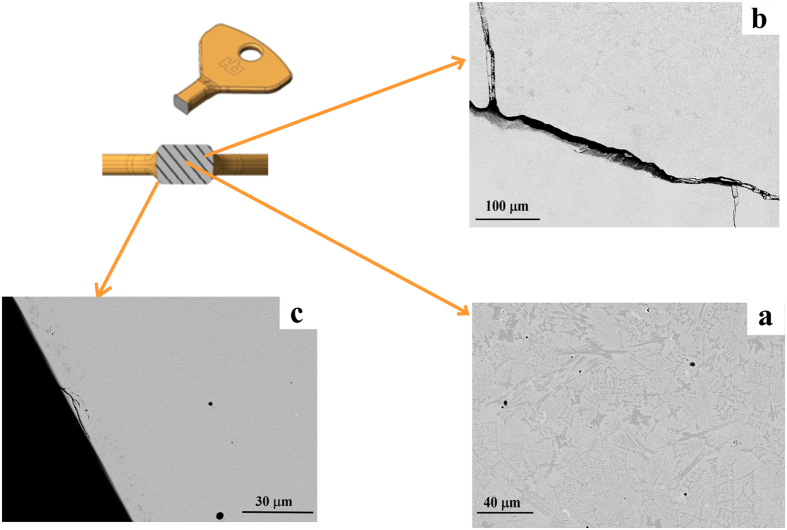
SEM images of the key cast at 1353 K (**a**) taken at the core of the key, showing a completely crystalline region; (**b**) taken in between the core and the outer surface, revealing the interface between the glassy and crystalline parts and (**c**) taken close to the outer surface, showing a completely glassy part of the key.

Figure 6 depicts typical SEM image taken in back scattered mode (BSE) at different places over the cross-section of the key stem cast at 1353 K. The image presented in Fig. 6(a) was taken at the core of the key. It reveals the presence of micron-size crystals and fine gas holes^40^ in the glassy matrix. The image presented in Fig. 6(b) was taken slightly away from the core and shows the interface between the glassy and crystalline part of the specimen, separated by a crack. The initial development of the crack between the glassy matrix and the crystalline part maybe mainly due to the thermal mismatch strain developed during the cooling process^45^. The image taken close to the surface shows a completely glassy part of the sample (Fig. 6(c)).

**Figure 7:**
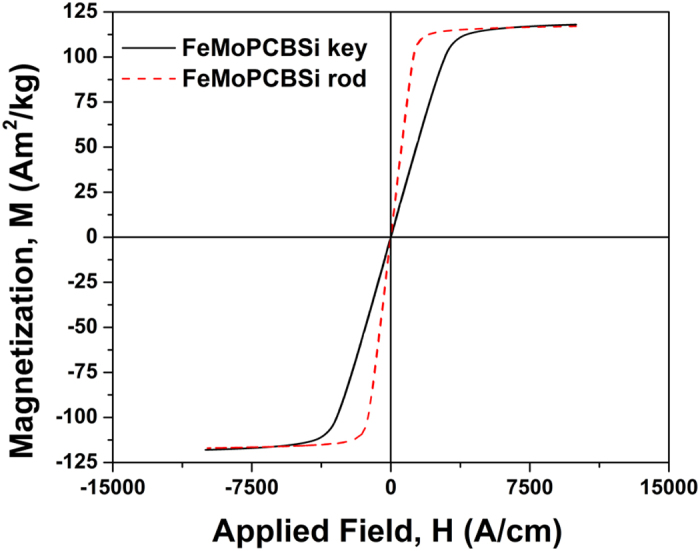
Hysteresis loops for a 2 mm rod cast under laboratory conditions and the key cast by HPDC.

The influence of the HPDC parameters on the magnetic properties was also investigated. Table 4 lists the variation of the coercivity (H_c_) of the keys as a function of flow rate (m/s), alloy temperature (in K) and die material along with the sample prepared in lab condition. The coercivity values support the earlier results, obtained from the DSC scans and the XRD patterns. The coercivity of the keys cast at low flow rate (4 m/s) using the steel die decreases from >2000 A/m to 7.8 A/m, as the casting temperature increases from 1353 K to 1573 K. Increasing the flow rate from 4 m/s to 19 m/s and using the same steel die causes a significant coercivity increase from 7.8 A/m to 80 A/m, indicating the presence of small crystals. In case of the Cu mold the coercivity decreases from >2000 A/m to 57.9 A/m for low flow rate. However, the samples cast at 1573 K show a relatively higher coercivity value (57.9 A/m), suggesting that the keys cast in the Cu die contain some crystals, in agreement with the previous findings. Figure 7 presents the hysteresis loops for the glassy rod cast in the laboratory condition (red dotted line) using conventional copper die casting method and the key cast with steel die (continuous line) at 1573 K using the HPDC method. The saturation magnetization of the samples are almost the same, lies within the experimental error.

## Discussion

The tool life has a large impact on the manufacturing costs of the products in industrial casting processes. To choose the most effective die material, a mechanically soft copper-based alloy with large heat conductivity (230 W/mK) and hardened heat resistant steel with small heat conductivity (33 W/mK) were selected for the present investigations (see Fig. 1(a,b)). During the experiments it was found that higher flow speed of the molten material and high casting temperature erode the copper-based die. In order to study the effect of the two die materials in more detail, an optimum flow rate of 4 m/s was chosen after several trials with different speeds. This flow rate was maintained constant by fixing the piston speed to 0.16 m/s. As it can be seen from the DSC results (see Fig. 2), the alloy temperature is also a very important parameter during the casting process. If the alloy temperature is just 50–100 K above the liquids temperature, nucleation starts before complete filling of the mold. The keys cast at 1353 K have the lowest first and second crystallization enthalpy values of −3.2 and −10.5 J/g, respectively. To achieve a homogeneous casting without flow lines and any other casting defects, the superheating of the alloy was slowly increased to over 250 K. At casting temperatures around 1573 ± 10 K very nice key samples were obtained (see Fig. 3a,b), with the first and second crystallization enthalpies reaching a maximum of −15.6 J/g and −38.8 J/g, respectively. Lin *et al*.^46^ systematically studied the effect of melt overheating prior undercooling on the increase in the oxygen percentage and therefore on the crystallization probability in Zr based BMGs. In their study they reported that overheating the melt above well-defined threshold temperature is required to achieve the maximum undercooling. Recently, Mukherjee *et al*.^47^ studied the effect of overheating up to 400 K in Zr based BMGs. They also observed a pronounced effect of overheating on the crystallization kinetics; they also observed that for every alloy there exists an overheating threshold above which the crystallization time increases. This increase in crystallization time may be due to the dissolution of the heterogeneities acting as a nucleation sites, if the overheating is less than the threshold limit the dissolution is incomplete and the nucleation starts quickly. Normally, the heterogeneities may arise due to minor traces of impurities, oxygen, high-temperature melting elements etc.; in our actual study we experimentally concluded that 1573 ± 10 K is the overheating threshold for this glass. This result is also supported by the XRD patterns (see Fig. 4a,b). The keys cast at the lower alloy temperatures of 1353 and 1393 K show relatively large crystalline peaks compared to the keys cast at 1443 K, which is almost 160 K above the liquidus temperature (*T*_*l*_ = 1283 K) of the alloy. In case of the steel die and a high casting temperature of 1573 K, the keys are completely amorphous. However, the keys cast using the copper die shows a decrease in crystallinity with increase in casting temperature, but still a small amount of crystals is observed in the core of the keys cast at 1573 K. This might be possibly due to the skin effect during HPDC^48^. The skin effect mostly occurs when the cooling rate of the cast part is very high; as a result the outer shell of the part solidifies very fast, leading to a gap between the sample and the die surface. The gap between die and cast part and amorphous outer shell hinders the heat transfer between the sample and the die, leading to nucleation of the crystals in the core of the sample^49^. It is well-known that in the melt-spinning process for the production of glassy structure, depending on the composition of the alloy, a Cu-wheel can outperform a steel wheel and vice versa. For example, to cast the ribbons with low surface roughness wheel materials with lower thermal conductivity *k* is preferred^50^. Lower the *k*, longer the dwell time between the substrate and molten metal which leads to a better surface roughness. Steel wheels are preferably used to prepare amorphous ribbons of soft magnetic alloys^51^ as well as several other binary alloys^52^. Hence, for casting of glassy keys the steel die is the most suitable choice, because of the lower cooling rate during solidification, better thermal contact, higher tool life and lower manufacturing cost. According to the literature^42^ the maximum achievable diameter reported for the investigated Fe_74_Mo_4_P_10_C_7.5_B_2.5_Si_2_ glass is 5 mm; in our work we were able to successfully cast plates with a maximum thickness of 3.5 mm (see Table 3). The extension of the supercooled liquid region (SLR, *ΔT*_*x*_ = *T*_*x*_ *−* *T*_*g*_) is 35 K and the reduced glass transition temperature *T*_*rg*_ = *T*_*g*_*/T*_*l*_ (0.57), are just slightly lower than the reported values of 37 K and 0.58, respectively^42^. Though a small difference in *T*_*x*_ and *T*_*g*_ values between the industrial cast key sample and lab cast rod samples is observed, they are well within the experimental error range. If the extension of the SLR and the reduced glass transition temperature are considered as important criteria to evaluate the GFA of the alloy^53^, then our alloy has a slightly lower GFA compared to the literature data. This decrease is GFA may be due to the difference in oxygen and impurity contents in the industrial raw materials. However, this small decrease is not significant as far as industrial casting processes are concerned.

In order to elucidate the influence of the material flow speed on the key quality, three different flow rates, i.e. 4, 12 and 19 m/s were applied. It was found that the increase in the flow speed decreases the surface quality and also increases the possibility of crystalline cores inside the keys. The increase in the metal flow rate from 4 to 19 m/s causes the crystallization enthalpies of the first and second crystallization events to decrease from −15.6 and −38.8 J/g to −9.8 and −26.7 J/g, respectively. This increase in crystallinity with increasing flow rate is believed to be due to the increase in shear rate of the melt at different cross-sections. Shao *et al*.^54^ in their recent studies showed the influence of shear rate in supercooled liquid state over the crystallization kinetics in Pt based BMG. The shear flow in the SLR influences the local compositional heterogeneities; with increase in shear flow the magnitude of the heterogeneities also increases leading to drastic decrease in crystallization time. Similar trend is observed in our casting as well, with increase in the metal flow rate from 4 to 19 m/s the crystallization rate increases. This may be because of the very high strain rate experienced by the molten metal while flowing through different cross-section in the die. The molten metal undergoes severe strain mainly while entering and exiting the shaft section, cross-section of the shaft is almost one fourth of the bit and head section. This repeated change in shear rate may induce several heterogeneous nucleation site, which leads to increases the fragility of the glass-forming liquid^44^. Moreover it is evident from the Flow 3D simulation analysis (see Fig. 5) that at higher material flow rates, i.e. 12 and 19 m/s, the mold filling is also poor, which leads to porosity, cold spots and poor surface finish. Hence, 4 m/s is an optimum flow rate to fill the complete mold without any defects and at this speed also the change in shear rate does not affect the GFA much.

The residual stresses developed during cooling play an important role in influencing the mechanical properties of the metallic glass^45^,^55^. Ustundag *et al*.^45^ have shown that residual stresses up to 900 MPa can be developed inside Zr-based bulk metallic glass during cooling. However, there is not enough data available for the magnitude of the residual stresses that may be present in the investigated Fe-based metallic glass, but apparently the stress generated is large enough to initiate cracks between the glassy and crystalline parts in the specimens. Once a crack is initiated it will further propagate and split into multiple branches in order to minimize the stress concentration^56^. The crack branching occurs predominantly at all possible interfaces between the glassy and crystalline parts of the specimens due to the differences in their mechanical/elastic properties^55^. The glass/crystal interface shown by the SEM image taken from the head of the key cast at 1353 K (see Fig. 6(b)) also reveals the skin effect. The skin effect is more visible in the keys cast at low temperatures using the copper die, but also exists in the keys cast at high temperature where the cores are very small and difficult to be detected by SEM. Hence, the coercivity of the samples was measured to make sure that the samples were completely amorphous. It is commonly accepted that soft magnetic BMG have very low values of coercivity^57^. If the BMG contains some small volume fraction of crystalline inclusions, the coercivity will increase significantly due to domain pinning. Only if the size of the crystals is comparable or smaller than the size of the domain walls, they have no influence on the coercivity^58^,^59^,^60^. Such small crystals or nuclei cannot be detected by X-ray diffraction. Although the samples cast under laboratory conditions and the ones obtained by HPDC exhibit the same saturation magnetization, there is some differences in the initial part of the loops. The coercivity and the shape of the hysteresis loop are greatly influenced by (a) the geometry and (b) the microstructure of the magnetic material^60^. The complex key geometry is a major factor influencing the magnetic properties of the key. With a proper geometry the size and shape effect could be reduced. Hence, with the above optimized parameters (casting speed, alloy temperature and die material) the HPDC method can be used to produce soft ferromagnetic BMG parts in complex geometries.

## Conclusions

In the present work, the effect of HPDC process parameters on microstructural evolution, thermal, and magnetic properties of soft magnetic Fe_74_Mo_4_P_10_C_7.5_B_2.5_Si_2_ BMG has been investigated. The quality of the samples is strongly influenced by proper choice of die material, alloy temperature and flow rate of the material. A copper die with high thermal conductivity leads to the skin effect. Similarly, a high flow of 19 m/s rate influences the viscosity change due to the higher shear rate leading to decrease in GFA of the glass. All samples produced under optimized conditions exhibit thermal stability and soft magnetic properties which are nearly the same as those of samples prepared under laboratory conditions. The great advantage of HPDC is that even marginal glass formers can be used and complex geometries with high dimensional accuracy can be achieved.

It should be mentioned here that the quality of the keys in the present work can be still optimized further by fine-tuning the casting parameters. This will improve the surface finish, reduce the porosity and hamper the formation of cracks during solidification. Nevertheless, these first results confirm that HPDC is useful for the production of bulk glassy alloys with good soft magnetic properties.

## Additional Information

**How to cite this article**: Ramasamy, P. *et al*. High pressure die casting of Fe-based metallic glass. *Sci. Rep.* **6**, 35258; doi: 10.1038/srep35258 (2016).

## Figures and Tables

**Table 1:** Comparision between HPDC, TPF and SLM processes.

HPDC process	SLM method process	TPF method process
Single step process	Multiple step process (samples are built layer by layer in several steps)	Multiple step process (Two or more stages are involved in the processes)
Casting process completes fast (high production rate)	Processing time mainly depending on the shape and size of the final product	Final shape decides the processing time (since the processing time is limited, it may take several heating cycles to complete the product^33^)
Widely studied and casting parameters well established (standard casting parameters are readily available for several alloy systems)	Standard parameters are available for few basic alloy systems only (standards are still in development stage^61^)	Only few alloys were processed by this method (needs development of more novel techniques to establishing standards)
Marginal glass formers can also be produced by this method (Due to application of high pressure good contact is established between the mold and the molten metal, which increases the cooling rate)	Only good glass formers with high thermal stability can be prepared by this method (during the addition of every new consecutive layer previous layer temperatures are subjected to increase in temperature, which leads to crystallization)	Only very good glass formers with high SLR can be processed (Good glass formers often contains toxic (Be^7^) or expensive elements like Pt, Pd, Au et.^62^,^63^)
Preparation of starting material is easy (normally induction melting or arc melting is preferred)	Difficult to get homogeneous multicomponent amorphous powder (SLM uses powder and the quality of the product depends on the quality of the powder^37^)	Glassy plate, rod or granules serves as starting material (quality of the glassy sample decides the final product quality)
Casting defects such as gas pockets and shrink holes can be drastically reduced or controlled^64^ (final product will have high density and low shrinkage)	Very difficult to avoid micro-pores and micro cracks (initial powder particle size and scanning speed plays important role in controlling the micro-pores and micro-cracks^37^,^38^)	During the process micro-pores and micro-cracks will be closed
Casting environment can influence the crystallization kinetics (casting die temperature, casting speed and casting atmosphere affect the final product)	Needs high purity inert atmosphere to produce the samples (The fine powders will oxidize or explode if the working atmosphere has oxygen)	TPF can be done in open atmosphere^65^
Samples have huge internal stress (the fast cooling results in large internal stress)	By pre-heating the base plate the internal stress can be reduced	Internal stress can be relieved by slow cooling of the sample after final processing step^35^
Limited reusability of the material (repeated usage leads to absorption of more oxygen, which influences the heterogeneous nucleation sites)	Powders can be reused after an additional sieving (The whole process is carried under inert atmosphere)	samples can be used after re -melting
Thin sections and sections with interconnected holes cannot be produced (viscosity of the molten alloy determines the minimum section thickness)	Any complex shape can be produced, powder size and the layer thickness are the only limiting factor, the technique has virtual no limitation	Thin and micro sections can be produced easily and easier than thick sections

**Table 2:** Enthalpies of the first and second crystallization step for the key samples cast at different temperatures using copper and steel dies.

Sample cast conditions			First Crystallization enthalpy (J/g) ±0.2	Second Crystallization enthalpy (J/g) ±0.2
Die material	Flow rate (m/s)	Casting Temperature (K) ±10		
Copper	4	1353	−3.2	−10.4
Copper	4	1393	−9.8	−28.7
Copper	4	1453	−11.8	−30.1
Copper	4	1573	−13.4	−32.8
Steel	4	1353	−3.8	−10.2
Steel	4	1393	−10.7	−27.5
Steel	4	1453	−12.4	−32.8
Steel	4	1573	−15.6	−38.8
Steel	12	1573	−13.1	−31.9
Steel	19	1573	−9.8	−26.7
Rod cast in lab - Copper die	—	1523	−15.8	−39.1

Enthalpies of the 3 mm rod cast in lab condition is given for reference purpose.

**Table 3:** Comparison between literature data (4 mm)^42^ and our data for the Fe_74_Mo_4_P_10_C_7.5_B_2.5_Si_2_ glassy samples (3 mm rod and key sample).

Fe_74_Mo_4_P_10_C_7.5_B_2.5_Si_2_	D (mm)	*T*_*g*_ [K]	*T*_*x*_ [K]	*ΔT*_*x*_ [K]	*T*_*l*_ [K]	*T*_*rg*_
Literature	4	729	766	37	1266	0.58
Key sample	—	725	760	35	1283	0.57
Sample cast in lab using cu mold	3	730	765	35	1283	0.57

*T*_*g*_ is the glass transition temperature, T_x_ is the onset of crystallization, *ΔT*_*x*_ the extension of the supercooled liquid region (SLR), measured as the difference between crystallization and glass transition temperatures, *T*_*l*_ is the liquidus temperature, *T*_*rg*_ the reduced glass transition temperature, measured as the ratio between the glass transition and liquidus temperatures.

**Table 4:** Coercivity of the keys as a function of casting speed (m/s), alloy temperature (K) and die material.

Die Material	Flow rate (m/s) ±0.5	Alloy Temperature (K) ± 10	Coercivity H_c_ (A/m) ±0.1
Steel	4	1573	7.8
Steel	4	1443	210
Steel	4	1393	>2000
Steel	4	1353	>2000
Steel	12	1573	9.8
Steel	19	1573	80
Copper	4	1573	57.9
Copper	4	1443	59.2
Copper	4	1393	>2000
Copper	4	1353	>2000
Rod cast in lab - Copper die	—	1523	5

Coercivity of the 3 mm rod cast in lab condition is given for reference purpose.
